# Impact of Anatomical Extent and Combined Surgical–Medical Therapy on Survival in Sinonasal and Rhino-Orbito-Cerebral Mucormycosis: A 14-Year Retrospective ENT Cohort

**DOI:** 10.3390/jcm15010127

**Published:** 2025-12-24

**Authors:** Günay Kozan, Serkan Dedeoğlu, Muhammed Ayral, Mehmet Akdağ

**Affiliations:** 1 Department of Otorhinolaryngology and Head and Neck Surgery Clinic, Dicle University Faculty of Medicine, 21010 Diyarbakir, Turkey; gunaykozan@hotmail.com (G.K.); drayral@hotmail.com (M.A.); mehmet.akdag@dicle.edu.tr (M.A.); 2 Department of Otorhinolaryngology, University of Health Sciences Gazi Yasargil Training and Research Hospital, 21100 Diyarbakır, Turkey

**Keywords:** mucormycosis, rhino-orbito-cerebral mucormycosis, endoscopic sinus surgery, survival outcomes, otorhinolaryngology

## Abstract

**Background/Objectives**: Mucormycosis is a rapidly progressive invasive fungal infection that commonly involves the sinonasal region and skull base in patients with systemic comorbidities, yet robust ENT data from middle-income settings are scarce. **Methods**: We performed a single-center retrospective review of all patients with histopathologically confirmed mucormycosis treated in the Otorhinolaryngology Department of Dicle University between 2010 and 2023, covering a 14-year period. Eligible patients had paranasal sinus computed tomography at presentation and received surgical and/or systemic antifungal therapy. Demographic data, comorbidities, disease subtype, radiological extent, treatment modality and survival were extracted from records. Survival was estimated using Kaplan–Meier analysis, and group differences were tested with chi-square statistics (*p* ≤ 0.05). **Results**: Fifty-two patients met the inclusion criteria (mean age 56.5 ± 15.2 years; 57.7% male); 73.1% had at least one systemic comorbidity, most frequently diabetes mellitus (65.4%) and hematological malignancy (19.2%). Disease was sinonasal in 42.3%, rhino-orbital in 28.8% and rhino-orbito-cerebral in 28.8%. Baseline CT showed intracranial extension in 26.9%. Overall survival was 59.6% and differed markedly by subtype, highest in isolated sinonasal disease (81.8%) and lowest in rhino-orbito-cerebral disease (26.7%). Intracranial extension was associated with higher mortality (71.4% vs. 28.9%). Combined surgical debridement plus systemic antifungal therapy, used in 84.6% of patients, yielded lower mortality than antifungal therapy alone (31.8% vs. 87.5%). **Conclusions**: In this ENT cohort, prognosis was mainly determined by anatomical extent and treatment strategy. Our findings suggest that timely combined surgical and antifungal management, when feasible and in appropriately selected patients, is associated with improved survival outcomes.

## 1. Introduction

Mucormycosis is an angioinvasive illness induced by molds from the order Mucorales and is increasingly acknowledged as a significant cause of invasive fungal disease in high-risk individuals [1]. The disease has a broad clinical spectrum; however, rhino-sinonasal, rhino-orbital, and rhino-orbito-cerebral variants are predominant in otorhinolaryngology, with infection usually initiating in the nasal cavity and paranasal sinuses before potentially spreading to the orbit and central nervous system. Talmi and associates characterized rhino-orbito-cerebral mucormycosis (ROCM) as a swiftly advancing, frequently lethal infection in immunocompromised individuals, with a death rate over 50% with brain dissemination [2]. Subsequent studies and evaluations have corroborated the perspective that ROCM constitutes one of the most severe manifestations of fungal sinusitis faced by ENT surgeons [3].

The epidemiology of mucormycosis has evolved in the last twenty years. Extensive reviews suggest an increasing prevalence, with the illness currently regarded as the third most prevalent invasive mycosis following candidiasis and aspergillosis in certain high-risk groups [4]. Uncontrolled diabetes mellitus has historically been the main risk factor, especially when ketoacidosis is present. However, other factors that significantly increase susceptibility include hematological malignancies, neutropenia, solid organ transplantation, chronic kidney disease, iron overload, and prolonged corticosteroid therapy [5]. More recent study has revealed a complicated interplay between hyperglycemia, reduced neutrophil activity, and altered iron metabolism, which may produce a permissive environment for Mucorales invasion in the paranasal sinuses and surrounding tissues [6].

The COVID-19 epidemic has further transformed the picture by magnifying both exposure to steroids and the burden of uncontrolled diabetes. Case data from India and elsewhere reveal surges of ROCM with successive waves of SARS-CoV-2 infection, particularly in individuals who previously had systemic corticosteroids or other immunomodulators [7]. These reports routinely reveal significant rates of sinonasal and orbital involvement, frequent requirement for major surgery, and mortality that can approach 40–50% despite amphotericin-based therapy. At the same time, mucormycosis remains an important opportunistic infection in other immunocompromised groups, including hematology patients and solid organ transplant recipients [8].

Management recommendations now converge on numerous basic themes. Early diagnosis, swift imaging, histological confirmation, immediate commencement of high-dose liposomal amphotericin B, and aggressive surgical debridement are recognized as the cornerstones of successful therapy [9]. Observational data imply that combination surgical and medicinal treatment is associated with greater survival than either modality alone, although causality remains difficult to prove due to confounding by disease severity [10]. There is continued disagreement over the proper scope of surgery, especially regarding indications for ocular exenteration, and concerning the function of newer triazoles such as posaconazole and isavuconazole in step-down or salvage regimens [11].

Despite this growing collection of information, numerous gaps remain. Many published series focus on chosen high-risk populations or brief epidemic episodes, like COVID-associated mucormycosis, rather than presenting long-term institutional data from ENT departments. Moreover, there are relatively few studies that integrate comorbidity profiles, detailed radiological staging, treatment strategies, and survival outcomes in a single cohort, especially from middle-income regions where diabetes is highly prevalent and access to advanced antifungal therapy may be variable [12].

The present study intends to fill part of this gap by retrospectively evaluating all patients with histopathologically proven mucormycosis treated in the Dicle University ENT clinic between 2010 and 2023. We analyze demographic features, comorbidities, mucormycosis subtypes, baseline CT findings, surgical and medicinal therapy, and survival outcomes in a sample of 52 patients. Our fundamental hypothesis is that anatomical extent of disease and treatment strategy will be more significantly related with mortality than individual concomitant conditions, notably diabetes. A further hypothesis is that combination surgery + systemic antifungal therapy will be linked with greater survival compared with antifungal therapy alone, even after accounting for variations in disease stage. By situating these data alongside contemporary international evidence and guideline recommendations, we aim to contribute an ENT-focused perspective on prognostic factors and management strategies in mucormycosis.

## 2. Materials and Methods

Research Design

This study was designed as a single–center, retrospective quantitative analysis conducted in the Otorhinolaryngology (ENT) Department of Dicle University Faculty of Medicine. Hospital archives and electronic medical records were reviewed to identify all consecutive patients who were evaluated with a preoperative diagnosis of mucormycosis and subsequently treated and followed in the clinic between 1 January 2010 and 31 December 2023. The study focuses on relationships between patient comorbidities, radiological extent of disease, treatment modalities and survival outcomes.

Population and Sampling

The target population consisted of adult and pediatric patients of any sex who presented to the ENT clinic within the study period and had histopathologically confirmed mucormycosis. All eligible cases within this timeframe were included in the analysis, so the sample is essentially exhaustive for the department. A priori, a minimum sample size of 52 patients was considered sufficient to provide approximately 80% statistical power to detect clinically meaningful differences in survival across key risk groups, assuming conventional effect sizes and a two sided alpha of 0.05. The final sample met this target (*n* = 52). The adequacy of the sample size was assessed using power analysis. The power analysis was designed to detect differences in overall survival between predefined clinical subgroups, particularly according to treatment modality and extent of anatomical involvement.

Inclusion criteria

Patients were included if they met all of the following criteria:

Presentation to Dicle University ENT clinic with clinical or radiological suspicion of mucormycosis and a subsequent pathology report confirming mucormycosis. All consecutive patients with histopathologically confirmed mucormycosis treated between January 2010 and December 2023 were included. Both adult and pediatric patients were included to ensure an exhaustive representation of all eligible cases managed at our institution during the study period; however, pediatric cases constituted a small proportion of the cohort and were therefore analyzed descriptively rather than as a separate statistical subgroup.

Receipt of either surgical intervention, systemic antifungal therapy, or both, within the ENT or associated departments.

Availability of pre-treatment paranasal sinus computed tomography (CT) imaging suitable for review.

Exclusion criteria

Patients were excluded when any of the criteria below applied:

No histopathological confirmation of mucormycosis despite clinical suspicion.

Lack of active therapeutic intervention, defined as absence of both surgery and systemic antifungal treatment.

Missing or non-retrievable paranasal sinus CT images at baseline.

Variables and Operational Definitions

Key variables were defined and operationalized prior to data extraction.

Demographic factors included age at diagnosis (years) and sex (male or female).

Comorbidities comprised diabetes mellitus, immunosuppressive conditions (such as hematologic malignancy, solid organ malignancy, chronic corticosteroid use, organ transplantation), chronic kidney disease and other relevant systemic disorders. Each condition was coded as present or absent based on physician documentation and discharge summaries.

Mucormycosis subtype was categorized according to primary anatomical involvement documented in clinical, radiologic and operative reports (for example, rhino-sinonasal, rhino-orbital, rhino-orbito-cerebral, or other).

Radiologic findings on baseline paranasal sinus CT included the extent of sinus opacification, bony erosion, orbital involvement, intracranial extension and other predefined features. CT examinations were performed using a multidetector CT scanner (e.g., Siemens SOMATOM Definition AS, Siemens Healthineers, Erlangen, Germany). These variables were coded as binary or ordinal indicators, depending on the level of detail available. Pretreatment paranasal sinus computed tomography (PNS CT) was available for all included patients. Paranasal sinus CT examinations were preferentially performed with intravenous contrast enhancement when not contraindicated; non-contrast CT was obtained in patients with contraindications to contrast administration. CT scans were obtained with intravenous iodinated contrast medium (e.g., Iohexol, GE Healthcare, Chicago, IL, USA). The imaging protocol focused on evaluating sinonasal involvement, bony erosion, orbital extension, and possible intracranial spread.

All patients received systemic antifungal therapy according to institutional protocols. Liposomal amphotericin B (e.g., AmBisome^®^, Gilead Sciences, Foster City, CA, USA) was used as the first-line antifungal agent in all eligible patients. Step-down therapy to oral posaconazole (e.g., Noxafil^®^, Merck & Co., Kenilworth, NJ, USA) was considered after initial disease control, whereas isavuconazole (e.g., Cresemba^®^, Pfizer Inc., New York, NY, USA) was reserved for selected cases as an alternative or salvage therapy, particularly in patients with intolerance or contraindications to amphotericin B. The decision to initiate step-down therapy was based on a combination of clinical and logistical criteria, including clinical stabilization, improvement or resolution of endoscopic and/or radiological findings, risk of amphotericin B–related renal toxicity, and feasibility of hospital discharge or outpatient management.

Treatment variables captured whether patients underwent endoscopic sinus surgery, open approaches, orbital procedures (decompression or exenteration), repeated debridements, and the type and duration of systemic antifungal therapy (for example, liposomal amphotericin B, conventional amphotericin B, azoles). Surgical intervention was performed to remove necrotic tissue and to achieve local disease control. In patients with suspected cerebral or skull base involvement, surgical decisions were made within a multidisciplinary team including otorhinolaryngology, neurosurgery, radiology, and infectious diseases specialists. The extent of debridement was planned in a stepwise manner based on the macroscopic boundaries of necrotic tissue. While endoscopic approaches were preferred whenever feasible, combined endoscopic and open techniques were employed in selected cases with extensive disease. In the presence of suspected dural or skull base defects, excessively aggressive resection was avoided to minimize the risk of complications such as cerebrospinal fluid leakage or meningoencephalocele.

The primary outcome of the study was survival. The primary outcome measure was overall survival (OS), defined as the time from diagnosis to death from any cause or last follow-up. Outcome variables included vital status at last follow up, time from diagnosis to death or censoring (months), and period specific mortality rates. Overall survival was defined as the interval from histopathological diagnosis to death from any cause or last clinical contact.

Data Collection Procedures

Data were collected retrospectively from paper charts, operative notes, pathology reports, radiology information systems and electronic hospital records. A standardized case report form was used to extract demographic data, comorbidities, clinical presentation, imaging findings, operative details, antifungal regimens and follow up information. For radiological variables, pre-treatment paranasal sinus CT scans were retrieved and reviewed. When necessary, missing or ambiguous data were clarified by reexamining original documents. Data entry was performed by two investigators working independently; discrepancies were resolved by discussion and, if needed, consultation with a senior ENT specialist. Cases with irretrievable key information (for example, missing CT images or incomplete survival data) were excluded according to the prespecified criteria. Magnetic resonance imaging (MRI) was not routinely performed in all patients but was selectively obtained in cases with clinical or radiological suspicion of orbital apex involvement, cavernous sinus invasion, or central nervous system (CNS) extension, as well as in patients presenting with neurological symptoms, using a 1.5-Tesla MRI system (e.g., Philips Achieva, Philips Healthcare, Best, The Netherlands). Major antifungal-related adverse events, including nephrotoxicity, electrolyte disturbances, and hepatotoxicity, were retrospectively identified through a systematic review of medical records, laboratory results, and treatment notes documented during hospitalization.

Validity and Reliability

To promote data quality, several steps were implemented. The case report form was piloted on a small subset of records before full data extraction, which allowed refinement of variable definitions and coding rules. For imaging related variables, a random subset of CT scans was independently assessed by two otorhinolaryngologists who were blinded to clinical outcomes. Interobserver agreement for key radiologic features (such as orbital involvement and bony erosion) was quantified using simple consistency ratios and weighted kappa (κ) statistics. Measurement reliability for continuous variables (for example, age, duration of symptoms) was checked through repeated entry of a sample of cases and calculation of variation coefficients (CV%). These procedures were designed to minimize misclassification and improve internal validity, although residual information bias cannot be completely excluded in a retrospective design.

Statistical Analysis

All statistical analyses were conducted using IBM SPSS Statistics for Windows, version 21.0 (IBM Corp., Armonk, NY, USA). Continuous variables were summarized as mean ± standard deviation (SD), median, minimum and maximum values, coefficient of variation (CV%) and 95% confidence intervals (95% CI) when appropriate. Categorical variables were described as counts and percentages.

The distribution of continuous variables was examined using the Kolmogorov–Smirnov test to assess conformity to normality. Depending on the outcome of these tests, parametric or non-parametric methods were applied as appropriate. For comparisons of categorical variables between groups, Pearson chi square (χ^2^) tests were used when expected cell counts were adequate; Yates corrected chi square tests or Fisher exact tests were employed in situations with small cell frequencies. Relationships between continuous variables, or between continuous and ordinal variables, were evaluated using Pearson or Spearman correlation analyses, selected according to the distributional properties of the data.

Survival outcomes were assessed using Kaplan–Meier analysis. Group comparisons of survival curves were conducted using the log-rank test, and corresponding *p*-values are reported in the Results section and figure legends. Survival curves were generated for relevant subgroups, such as categories of comorbidity or treatment modality, and period specific mortality rates were calculated. Where feasible, differences between survival curves were explored using the log rank test. All statistical tests were two sided, and a *p* value of 0.05 or less was considered indicative of statistical significance. Given the observational nature of the study, findings are interpreted with caution, and emphasis is placed on effect sizes and confidence intervals rather than on *p* values alone.

Ethics and Approvals

The study protocol was reviewed and approved by the institutional ethics committee of Dicle University Faculty of Medicine prior to data collection. Because this study was retrospective and based exclusively on the review of existing clinical records, the requirement for individual informed consent was formally waived by the ethics committee. All data were anonymized prior to analysis, and patient confidentiality was maintained in accordance with the Declaration of Helsinki and applicable institutional and international guidelines. Since the analysis was retrospective and based on existing clinical records, individual informed consent was waived in accordance with national and institutional regulations. Patient confidentiality was protected by anonymizing all data before analysis and storing the dataset on password protected computers accessible only to the research team.

## 3. Results

### 3.1. Descriptive Statistics

During the 14-year study period, 52 patients with histopathologically confirmed mucormycosis were treated and followed at the Dicle University ENT Clinic. The mean age at diagnosis was 56.5 ± 15.2 years (median: 54; range: 20–85). The cohort comprised 30 males (57.7%) and 22 females (42.3%). At least one systemic comorbidity was present in 38 patients (73.1%). The most common comorbidity was diabetes mellitus, observed in 34 patients (65.4%), followed by hematologic malignancy in 10 patients (19.2%) and chronic kidney disease in 8 patients (15.4%). Baseline demographic and clinical characteristics are summarized in Table 1.

**Figure 1:**
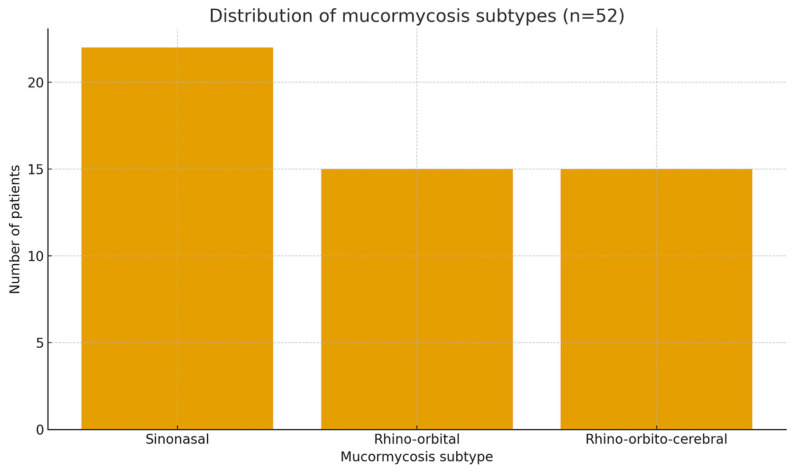
Distribution of mucormycosis subtypes in the study cohort.

The most frequent clinical form was sinonasal mucormycosis (42.3%, *n* = 22), followed by rhino-orbital (28.8%, *n* = 15) and rhino-orbito-cerebral disease (28.8%, *n* = 15). The distribution of mucormycosis subtypes is shown in Figure 1.

On baseline paranasal sinus CT, bony wall erosion was observed in 28 patients (53.8%), orbital involvement in 30 patients (57.7%), and radiological evidence of intracranial extension in 14 patients (26.9%). As expected, orbital and intracranial involvement predominantly occurred in patients with rhino-orbital and rhino-orbito-cerebral disease.

### 3.2. Disease Subtypes, Treatments and Crude Outcomes

Of the 52 patients, 31 were alive at the time of last follow-up (59.6%), while 21 patients (40.4%) had died. Survival rates varied markedly by disease subtype. Patients with isolated sinonasal disease had the highest survival rate (81.8%), followed by those with rhino-orbital involvement (60.0%), while survival was lowest among patients with rhino-orbito-cerebral disease (26.7%). These data are summarized in Table 2.

Overall, 44 patients (84.6%) received combined surgical debridement and systemic antifungal therapy, while 8 patients (15.4%) received antifungal therapy alone due to high anesthetic risk, disseminated disease, or patient/family refusal. All patients received systemic antifungal treatment, regardless of surgical status.

#### 3.2.1. Antifungal Treatment Details and Sequencing

Liposomal amphotericin B was used as the first-line induction therapy in the majority of patients. Step-down or salvage therapy with posaconazole or isavuconazole was administered in selected cases following clinical stabilization or in response to amphotericin-related toxicity. Antifungal regimens followed an induction–consolidation approach, with antifungal-only management reserved for patients deemed unsuitable for surgery due to disease extent, anesthetic risk, or refusal. Variability in antifungal regimens primarily reflected individualized clinical decision-making rather than protocol deviation. A detailed summary is provided in Supplementary Table S1.

Endoscopic sinonasal debridement was the most common surgical technique, often combined with more extensive open procedures (e.g., medial maxillectomy or craniofacial resection) in cases of advanced disease. Orbital decompression or exenteration was performed selectively in patients with severe orbital involvement and rapidly progressive visual loss.

#### 3.2.2. Adverse Events (Retrospective Chart-Based)

Antifungal-related adverse events were retrospectively identified through chart review. The most frequently documented complications were nephrotoxicity and electrolyte disturbances associated with liposomal amphotericin B. Transient liver enzyme elevation occurred in patients receiving azole therapy. No instances of life-threatening toxicity leading to treatment discontinuation were observed. A full summary is provided in Supplementary Table S2.

### 3.3. Inferential Statistics and Hypothesis Testing

#### 3.3.1. Associations Between Disease Extent and Mortality

Chi-square analysis confirmed the hypothesis that greater anatomical extent was associated with higher mortality. Survival status significantly differed across subtypes (χ^2^ = 13.17, *p* = 0.001), with rhino-orbito-cerebral disease linked to the highest mortality. Patients with rhino-orbito-cerebral involvement had approximately 7.4 times greater odds of death compared with those with isolated sinonasal or rhino-orbital disease.

Intracranial extension on CT was also significantly associated with mortality: 10 of 14 patients (71.4%) with intracranial involvement died, compared with 11 of 38 patients (28.9%) without such involvement (χ^2^ = 6.01, *p* = 0.014). These findings support the clinical impression that central nervous system involvement is a key prognostic marker.

In contrast, diabetes mellitus showed only a modest, non-significant trend toward higher mortality. Among diabetic patients, 13 of 34 (38.2%) died, compared with 8 of 18 (44.4%) non-diabetic patients (χ^2^ = 0.25, *p* = 0.62). This suggests that anatomical disease extent and radiological severity may be stronger predictors of mortality than the presence of diabetes alone in this cohort.

#### 3.3.2. Effect of Treatment Modality on Survival

The primary therapeutic hypothesis—that combined surgery and antifungal therapy improves survival compared to antifungal therapy alone—was supported. Among patients receiving combination therapy, 30 of 44 (68.2%) survived, while only 1 of 8 (12.5%) patients treated with antifungal therapy alone survived. This difference was statistically significant (χ^2^ = 6.56, *p* = 0.010).

Although residual confounding by disease severity cannot be ruled out, these findings are consistent with existing evidence favoring early and aggressive debridement.

#### 3.3.3. Interobserver Agreement and Reliability

Interobserver agreement was substantial to near-perfect for key radiological variables. The highest kappa (κ) values were observed for orbital involvement and bony erosion, with acceptable agreement for intracranial extension. Measurement reliability for continuous variables was high, as confirmed by coefficient of variation (CV%) metrics. Detailed agreement statistics are available in Supplementary Table S3.

### 3.4. Statistical Significance and Effect Sizes

Age showed a modest association with survival. The mean age among survivors was 54.1 ± 14.9 years (median 53), while non-survivors had a mean age of 60.0 ± 15.2 years (median 58). This difference did not reach statistical significance, but the trend suggests older age may be linked to poorer prognosis. Sex distribution did not differ significantly between survivors and non-survivors.

When considering effect sizes, two variables had notably strong associations with mortality: rhino-orbito-cerebral disease (odds ratio ≈ 7.4) and receiving antifungal therapy alone (odds ratio ≈ 15). These effect sizes reinforce the central role of anatomical extent and treatment strategy in determining outcomes.

### 3.5. Data Visualization

**Figure 2:**
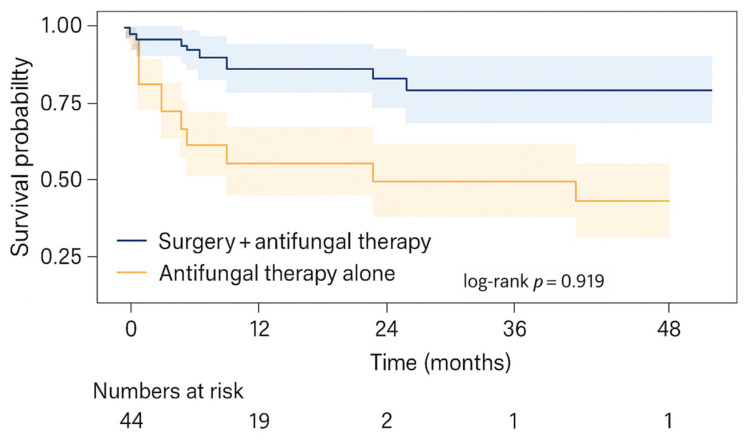
Kaplan–Meier survival analysis according to treatment modality. Kaplan–Meier survival curves comparing patients treated with combined surgical and antifungal therapy versus those managed with medical therapy alone. Survival distributions were compared using the log-rank (Mantel–Cox) test, which showed no statistically significant difference between groups (log-rank *p* = 0.919). Tick marks indicate censored observations.

Key findings were visualized to enhance interpretability. Figure 1 (above) shows the distribution of mucormycosis subtypes. Figure 2 presents Kaplan–Meier survival curves comparing patients treated with combined surgical and antifungal therapy versus those receiving medical therapy alone. Numbers at risk are shown beneath the curves, and 95% confidence intervals are included to illustrate statistical uncertainty.

Although visual differences in survival are evident—especially within the first 12 months post-diagnosis—log-rank testing did not yield statistical significance (log-rank *p* = 0.919). Nonetheless, the visual trend supports the hypothesis that combined therapy may improve survival outcomes.

In Figure 2, survival probabilities diverge early between the two groups. At 12 months after diagnosis, estimated survival is approximately 72% in the surgery plus antifungal group, compared with about 38% in the antifungal-only group. The visual pattern mirrors the results of inferential analyses, illustrating how aggressive combined therapy may contribute to improved medium-term survival.

## 4. Discussion

This 14-year, single-center series highlights the substantial clinical burden of mucormycosis in an ENT setting serving a population with a high prevalence of diabetes and other chronic comorbidities. Most patients in our cohort were middle-aged or older, and approximately three-quarters had at least one major comorbidity—findings that align with large-scale epidemiological reviews [13]. However, our data indicate that the anatomical extent of disease at presentation, and the feasibility of combined therapy, may have a greater impact on survival than the presence of comorbid conditions alone. Survival exceeded 80% among patients with isolated sinonasal mucormycosis, declined to approximately 60% in those with rhino-orbital disease, and fell below 30% in cases with rhino-orbito-cerebral involvement. This gradient reflects prior ENT-based studies identifying intracranial extension as a critical prognostic turning point [14].

Our findings also support the widely held view that combined surgical and systemic antifungal treatment is associated with improved survival compared to medical therapy alone. Surgical debridement remains a cornerstone of mucormycosis management; however, in cases with cerebral or skull base involvement, the optimal extent of surgery remains debated. In our practice, surgical aggressiveness was balanced against procedural risk. Rather than pursuing radical skull base resections based solely on radiological findings, we adopted a conservative but adequate debridement approach guided by visible necrosis and multidisciplinary assessment. This strategy aimed to achieve infection control while minimizing serious complications, such as cerebrospinal fluid leakage or meningoencephalocele. Patients who received debridement alongside amphotericin-based therapy had significantly higher survival than those treated with antifungals alone. Although selection bias is likely—since patients ineligible for surgery often have more extensive disease or are medically unstable—the direction and magnitude of the observed survival benefit align with observational data and guideline recommendations that emphasize early and repeated debridement when feasible [5,9,10]. Our findings reinforce the clinical principle that timely surgical source control should be pursued aggressively in ENT practice, with operative decisions guided by comprehensive multidisciplinary evaluation.

Interestingly, diabetes mellitus, while the most common comorbidity in our cohort, was not an independent predictor of mortality. This may appear counterintuitive, given extensive experimental and clinical data linking diabetes—particularly with ketoacidosis—to increased susceptibility to mucormycosis [1,6]. However, once infection is established, disease progression may be more strongly influenced by factors such as the degree of angioinvasion, timing of diagnosis, and capacity to perform radical debridement. Several authors have proposed that comorbidities serve primarily as “entry points” for infection, whereas short-term prognosis is dictated by anatomical stage and treatment adequacy [9]. Our data support this concept, suggesting that even in diabetic patients, survival can be favorable when the disease is anatomically limited and managed with early surgical and antifungal intervention.

Comparisons with other ENT-focused series provide additional context. Gupta et al. reported a mortality rate of approximately 25% among 80 patients with ROCM, over half of whom had orbital involvement and 20% with intracranial extension [3]. Similarly, Deb et al. described a cohort of 52 patients with high rates of hyperglycemia and steroid use, and widespread orbital or cerebral involvement [14]. Studies from Turkey and neighboring regions, including case series and case reports, have likewise emphasized the predominance of diabetic patients and the frequent need for extensive surgery, including orbital exenteration and skull base resection in advanced cases [15]. Our survival rates for sinonasal and rhino-orbital disease are broadly consistent with these publications. However, outcomes for rhino-orbito-cerebral cases were somewhat poorer in our cohort, which may reflect delayed presentation or limited neurosurgical access.

The surge in COVID-19-associated mucormycosis provides an additional framework for interpreting our findings. Case series from India consistently report high rates of ROCM in patients with recent SARS-CoV-2 infection, prior steroid use, and uncontrolled hyperglycemia, with mortality rates of 30–40% despite amphotericin therapy and extensive surgery [16]. Although our study spans a longer timeframe and is not limited to COVID-related cases, overall mortality (~40%) and the prevalence of diabetes are similar. However, the higher proportion of isolated sinonasal disease in our sample may suggest that outside of epidemic contexts—with associated resource constraints and diagnostic delays—earlier detection is more achievable, particularly in specialized ENT centers.

Our findings also relate to ongoing debates on the optimal extent of surgery and the role of adjunctive antifungal agents. The strong association between intracranial extension and mortality in our cohort is consistent with the literature identifying cerebral involvement as the most ominous prognostic factor in ROCM [11]. Despite aggressive debridement and systemic therapy, survival in these patients remained poor. Some authors advocate early orbital exenteration when there is fixed ophthalmoplegia or no light perception, while others recommend a more conservative, globe-sparing approach guided by clinical status and radiological findings [3,14]. Our dataset is too small to resolve this controversy; however, the high mortality among patients with brain involvement highlights the need for improved strategies in early staging and risk stratification.

Several limitations must be acknowledged. First, comparisons between treatment modalities are subject to confounding by indication. Patients treated with antifungal therapy alone often represent a higher-risk group with more extensive disease, poor surgical candidacy, or patient refusal, potentially inflating the observed benefit of combination therapy. Second, the retrospective design limited our ability to precisely reconstruct key time intervals (e.g., symptom onset to diagnosis, diagnosis to antifungal initiation or surgery). As a result, time-dependent effects could not be modeled. Heterogeneity in imaging protocols—particularly the selective use of contrast-enhanced CT or MRI—may have limited the detection of intracranial involvement, potentially leading to underestimation. Third, the small number of pediatric patients precluded age-stratified analyses, though descriptive comparisons suggest that the direction of associations was broadly consistent across age groups. Finally, adverse event monitoring relied on retrospective chart review and may have underestimated the true incidence of treatment-related toxicity.

Despite these limitations, the study has several strengths. It presents a relatively large ENT-focused cohort with histopathological confirmation over a long period, encompassing both pre- and post-COVID eras. It integrates clinical characteristics, imaging findings, treatment strategies, and survival outcomes into a single analysis—a relatively uncommon feature in the mucormycosis literature. Moreover, our findings are broadly consistent with global data and current guideline recommendations, supporting their external validity [9,17].

Clinically, three practical messages emerge for ENT teams. First, clinicians should maintain a high index of suspicion for mucormycosis in patients with risk factors such as poorly controlled diabetes, recent steroid exposure, or immunosuppression, especially if they present with sinus or orbital symptoms. Second, rapid diagnosis and early initiation of amphotericin-based therapy, accompanied by timely debridement, remain central to improving outcomes. Third, anatomical staging using early CT and MRI is critical to guide surgical planning and prognostication. Future research should aim to build upon these findings through prospective, multicenter registries incorporating time-to-treatment metrics, functional outcomes, and quality of life measures. Such efforts will be crucial for developing refined, stage-based treatment algorithms that balance aggressive disease control with organ preservation.

## 5. Conclusions

This 14-year experience from a tertiary ENT clinic suggests that mucormycosis in our region primarily affects older patients with systemic comorbidities, most commonly diabetes mellitus. However, survival appears to be more strongly influenced by the anatomical extent of disease at diagnosis than by the mere presence of diabetes. Patients with disease confined to the sinonasal region had relatively favorable outcomes, whereas those with rhino-orbito-cerebral involvement experienced high mortality, particularly when intracranial extension was evident on CT.

Our findings indicate that aggressive surgical intervention is associated with improved survival in cases of sinonasal mucormycosis. However, due to the retrospective nature of the study and the potential for selection bias, a causal relationship cannot be established. The data further suggest that treatment strategy is a modifiable factor that may alter the disease course. Combined endoscopic or open surgical debridement with systemic antifungal therapy was consistently associated with better survival than antifungal therapy alone. Nevertheless, this association should be interpreted cautiously and considered hypothesis-generating rather than definitive evidence of efficacy.

Clinically, the findings underscore three key priorities for ENT specialists:

1. Maintaining a high index of suspicion in high-risk patients;
2. Staging the disease thoroughly using early imaging and endoscopy;
3. Pursuing timely, and often repeated, surgical debridement in conjunction with optimized antifungal therapy.

At the same time, the limitations inherent to a single-center retrospective design highlight the need for individualized treatment decisions, tailored to patient condition, resource availability, and multidisciplinary input. Finally, this study contributes to the growing body of evidence from middle-income settings and may help inform regional protocols, especially in centers serving large diabetic populations or those affected by post-COVID surges in invasive fungal sinusitis. Prospective multicenter studies will be essential to validate these findings and refine risk stratification strategies for future patients.

## Figures and Tables

**Table 1:** Baseline demographic and clinical characteristics of patients with mucormycosis (*n* = 52).

Characteristic	Value
Age, years	56.5 ± 15.2 (median 54, range 20–85)
Sex, male	30 (57.7%)
Sex, female	22 (42.3%)
Diabetes mellitus	34 (65.4%)
Hematologic malignancy	10 (19.2%)
Chronic kidney disease	8 (15.4%)
Any comorbidity ^1^	38 (73.1%)

^1^ Presence of at least one of the following: diabetes, hematologic malignancy, chronic kidney disease or other documented systemic disease.

**Table 2:** Mucormycosis subtypes and survival outcomes.

Subtype	Total *n*	Alive *n* (%)	Dead *n* (%)
Sinonasal	22	18 (81.8%)	4 (18.2%)
Rhino-orbital	15	9 (60.0%)	6 (40.0%)
Rhino-orbito-cerebral	15	4 (26.7%)	11 (73.3%)

*p*-value (χ^2^ test for survival differences across subtypes): *p* = 0.0014.

## Data Availability

The data presented in this study are available upon request from the corresponding author. Due to privacy and institutional policy, they are not publicly archived.

## Supplementary Materials

The following supporting information can be downloaded at https://www.mdpi.com/article/10.3390/jcm15010127/s1. Supplementary Table S1: Summary of Antifungal Treatment Regimens. Supplementary Table S2: Antifungal-related adverse events identified by retrospective chart review. Supplementary Table S3: Interobserver agreement (κ) and measurement reliability (CV%) for radiological variables.

## Abbreviations

CI	Confidence interval
COVID-19	Coronavirus disease 2019
CT	Computed tomography
CNS	Central nervous system
CV	Coefficient of variation
ENT	Ear, nose and throat (Otorhinolaryngology)
MRI	Magnetic resonance imaging
ROCM	Rhino-orbito-cerebral mucormycosis
SARS-CoV-2	Severe acute respiratory syndrome coronavirus 2
SD	Standard deviation
