# Supplementary material for: Impact of Anatomical Extent and Combined Surgical–Medical Therapy on Survival in Sinonasal and Rhino-Orbito-Cerebral Mucormycosis: A 14-Year Retrospective ENT Cohort
Source: J Clin Med. 2025 Dec 24;15(1):127. doi: 10.3390/jcm15010127 (PMC12787092; doi:10.3390/jcm15010127)
Supplement: Supplementary file 1 [file jcm-15-00127-s001.zip › jcm-4042803-supplementary.pdf]

Supplementary Table S1. Summary of Antifungal Treatment Regimens.

Treatment Component	Description
**First-line antifungal agent**	Liposomal amphotericin B
**Induction therapy**	Liposomal amphotericin B administered as initial induction therapy in the majority of patients
**Step-down / salvage therapy**	Posaconazole or isavuconazole used as step-down or salvage therapy in selected patients
**Indications for step-down therapy**	Clinical stabilization, completion of induction phase, or amphotericin B-related toxicity
**Treatment sequencing**	Induction (liposomal amphotericin B) → consolidation/maintenance (azole-based therapy)
**Overall treatment duration**	Variable, depending on clinical response, disease extent, and patient tolerance
**Antifungal-only management**	Reserved for patients unsuitable for surgery due to extensive disease, prohibitive anesthetic risk, or patient refusal
**Treatment heterogeneity rationale**	Reflects individualized clinical decision-making rather than protocol inconsistency

Supplementary Table S1. Summary of antifungal treatment regimens used in the study cohort. Antifungal therapy was individualized based on disease extent, clinical status, and treatment tolerance, generally following an induction–consolidation strategy.

Supplementary Table S2. Antifungal-related adverse events identified by retrospective chart review (n = 52).

Antifungal agent	Adverse event type	n (%)	Severity (CTCAE)	Management / Outcome
Liposomal amphotericin B (n = 48)	Nephrotoxicity (↑ serum creatinine)	9 (18.8%)	Grade 1–2	Dose adjustment and intravenous hydration; no permanent discontinuation
Liposomal amphotericin B (n = 48)	Electrolyte imbalance (hypokalemia and/or hypomagnesemia)	14 (29.2%)	Grade 1–2	Electrolyte replacement and monitoring
Posaconazole (n = 18)	Elevated liver enzymes (AST/ALT)	3 (16.7%)	Grade 1	Transient; resolved with continued therapy and monitoring
Isavuconazole (n = 6)	Elevated liver enzymes (AST/ALT)	1 (16.7%)	Grade 1	No treatment modification required
Any antifungal agent	Life-threatening toxicity	0 (0%)	–	Not observed

Abbreviations: AST, aspartate aminotransferase; ALT, alanine aminotransferase.
CTCAE: Common Terminology Criteria for Adverse Events (retrospective grading where feasible).

Supplementary Table S3. Interobserver agreement (κ) and measurement reliability (CV%) for radiological variables.

**Radiological variable**	**Kappa (κ)**	**Agreement level (Landis & Koch)**	**Repeated-measure CV (%)**
Orbital involvement	**0.82**	Almost perfect	**4.6**
Bony erosion	**0.79**	Substantial	**5.2**
Intracranial extension	**0.71**	Substantial	**6.8**

Abbreviations: κ, Cohen's kappa; CV, coefficient of variation.
